# Mesenchymal stem cells microvesicles stabilize endothelial barrier function partly mediated by hepatocyte growth factor (HGF)

**DOI:** 10.1186/s13287-017-0662-7

**Published:** 2017-09-29

**Authors:** Hualing Wang, Ruiqiang Zheng, Qihong Chen, Jun Shao, Jiangquan Yu, Shuling Hu

**Affiliations:** 1 Department of Cardiology, Subei People’s Hospital, School of Medicine, Yangzhou University, 98 Nantong West Road, Yangzhou, 225001 People’s Republic of China; 2 Department of Critical Care Medicine, Subei People’s Hospital, School of Medicine, Yangzhou University, 98 Nantong West Road, Yangzhou, 225001 People’s Republic of China; 3 Department of Critical Care Medicine, Zhongshan Hospital, Fudan University, Shanghai, 200032 China

**Keywords:** Mesenchymal stem cells microvesicles, Hepatocyte growth factor, Endothelial permeability, Acute lung injury

## Abstract

**Background:**

Mesenchymal stem cells microvesicles (MSC-MVs) stabilize endothelial barrier function in acute lung injury (ALI); however, the detailed mechanism remains to be further defined. Hepatocyte growth factor (HGF), which is derived from MSC-MVs, might have a key role in the restoration of endothelial barrier function by MSC-MVs.

**Methods:**

MSCs with lentiviral vector-mediated HGF gene knockdown (siHGF-MSC) were generated. A co-culture model of pulmonary microvascular endothelial cells and MSC-MVs collected from MSCs or siHGF-MSCs after 24 h of hypoxic culture was utilized. Then, endothelial paracellular and transcellular permeabilities were detected. VE-cadherin, and occludin protein expression in the endothelial cells was measured using Western blot. Endothelial cell proliferation was analysed by 3-(4,5-dimethylthiazol-2-yl)-2,5-diphenyltetrazolium (MTT) assay. Endothelial cell apoptosis was analysed using TUNEL assay. Finally, IL-6 and IL-10 production was determined via an enzyme-linked immunosorbent assay (ELISA).

**Results:**

Treatment with MSC-MVs significantly decreased LPS-induced endothelial paracellular and transcellular permeabilities, and the effect was significantly inhibited after HGF gene knockdown in MSC-MVs. Furthermore, treatment with MSC-MVs increased the expression of the endothelial intercellular junction proteins VE-cadherin and occludin. Treatment with MSC-MVs also decreased endothelial apoptosis and induced endothelial cell proliferation. Finally, the treatment reduced IL-6 production and increased IL-10 production in the conditioned media of endothelial cells. However, the effects of the treatment with MSC-MVs were inhibited after HGF gene knockdown.

**Conclusions:**

MSC-MVs protect the barrier functions of pulmonary microvascular endothelial cells, which can be partly attributed to the presence of HGF in the MSC-MVs.

## Background

Acute lung injury (ALI) is characterized by increased lung permeability, pulmonary oedema and diffuse inflammation and results in the disruption of alveolar-capillary membranes [1]. Pulmonary microvascular endothelial cells (PMVEC) serves as a semi-selective barrier between the plasma and interstitium to micromolecules and bioactive agents [2]. Many agents, such as bacterial lipopolysaccharides (LPS), lead to an increase in endothelial permeability by activating inflammatory responses, which contributes to the development of ALI. There are two pathways regulating permeability across the endothelial barrier, paracellular and transcellular [3]. Paracellular permeability is primarily determined by adherens junction (AJ) and tight junctions. In contrast, the transcellular pathway is defined as vesicle-mediated transport of macromolecules across the endothelial barrier in a caveolae-dependent manner [4]. Thus, stabilizing PMVEC barrier function is critical for treating ALI.

Previous studies have shown that mesenchymal stem cells (MSCs) improved endothelial permeability in ALI [5, 6]. Recently, MSCs have been found to release microvesicles (MSC-MVs), which can be involved in cell-cell communication and the transfer of microRNA, protein or other signalling molecules [7]. A study indicated that MSC-MVs had the same therapeutic effect as MSCs themselves in *E. coli* endotoxin-induced ALI in mice through the transfer of keratinocyte growth factor (KGF) microRNA, which decreased endothelial permeability [8]. Therefore, MSC-MVs have good prospects for treating ALI.

Our previous study has shown that hepatocyte growth factor (HGF) secreted by MSCs is a key factor associated with endothelial permeability [9]. HGF is present in the lung circulation under pathological conditions such as acute lung injury and exhibits continuous barrier protective effects on human pulmonary endothelial cells [10]. Studies have shown that the HGF mRNA present in MVs derived from stem cells was delivered into cells and translated into the HGF protein as a mechanism of HGF’s induction of cell differentiation and growth [11]. Thus, we assume that HGF derived from MSC-MVs may have a key role in the regulation of endothelial permeability by MSC-MVs.

The aim of the present study was to determine the effects and mechanisms of MSC-MVs on LPS-induced endothelial permeability. We investigated the effects of MSC-MVs on endothelial paracellular and transcellular permeabilities using in vitro co-culture experiments. We then explored the mechanisms by which MSC-MVs regulate endothelial permeability by knocking down HGF in MSC-MVs.

## Methods

### MSC culture

**Fig. 1:**
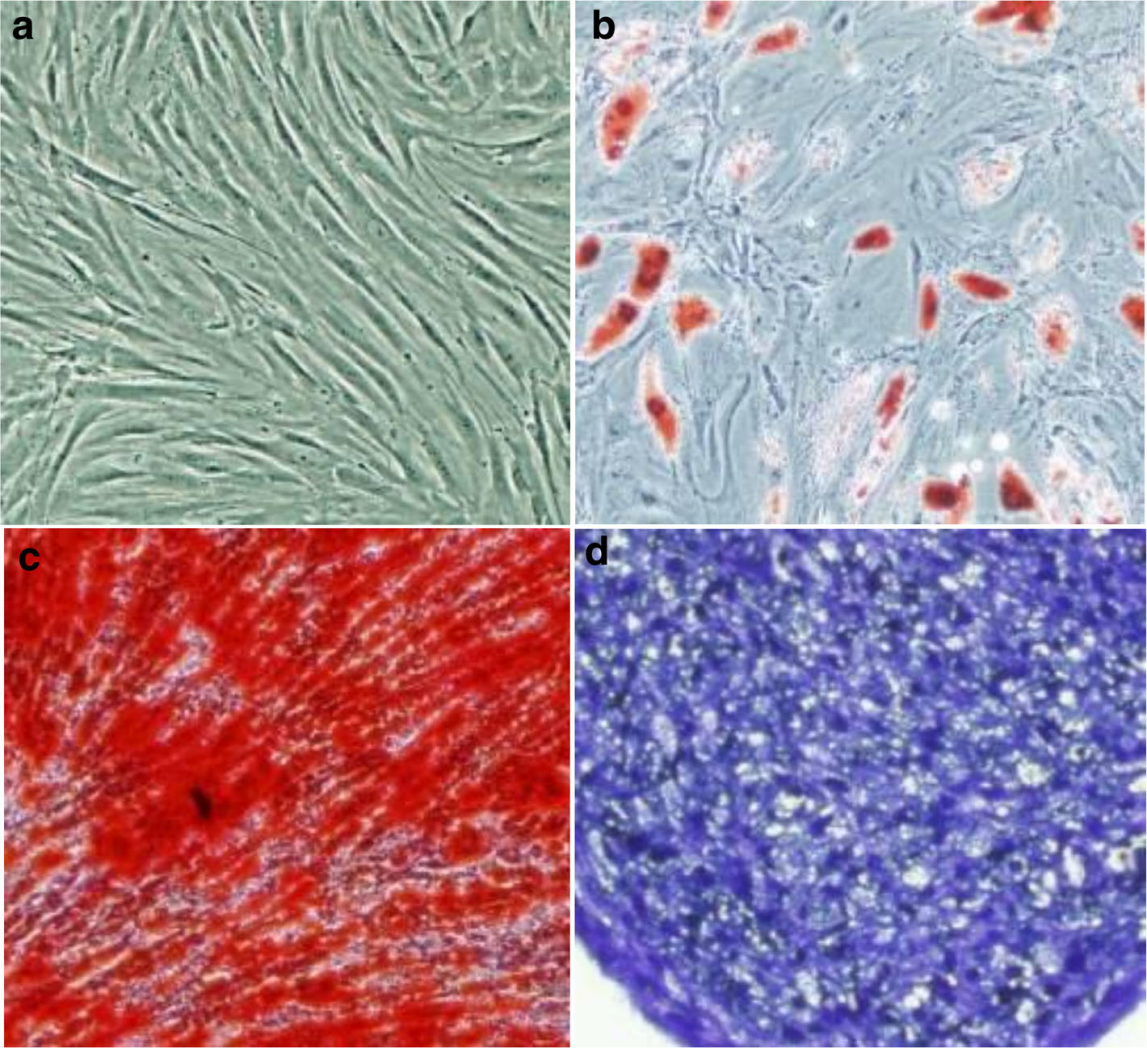
Multilineage differentiation identification of MSCs. The morphology of MSCs at the third passage (**a** × 100) and multilineage differentiation capacities of MSCs, including adipogenic differentiation stained with oil red-O (**b** × 200), osteogenic differentiation stained with alizarin red (**c** × 200), and chondrogenic differentiation stained with toluidine blue (**d** × 200), were observed with a microscope

Mice bone marrow-derived MSCs and mice pulmonary microvascular endothelial cells were used in the present study. MSCs were purchased from Cyagen Biosciences Inc. (Guangzhou, China). The cells were identified by detecting cell surface phenotypes by flow cytometry analyses as previously [9]. To verifying their identity as MSC, their multipotency for differentiation along with the adipogenic, osteogenic, and chondrogenic lineages were determined by staining with oil red-O, alizarin red, or toluidine blue, respectively, followed by culture in adipogenic, osteogenic, or chondrogenic differentiation media (Cyagen Biosciences Inc.) for 2–3 weeks (Fig. 1). The MSCs were cultured in MSC growth medium (Cyagen Biosciences Inc.). All the cells were cultured in a humidified 5% CO_2_ incubator at 37 °C. The culture media was changed every 3 days, and the cells were used at passages 3–7 for all experiments. MSCs with lentiviral vector-mediated HGF gene knockdown (siHGF-MSC) were generated as previously described [5].

### Isolation and characterization of MSC-MVs

MSC-MVs obtained from supernatants of MSCs were isolated by differential ultracentrifugation and characterized as described [12]. Briefly, the MSC-MVs were obtained from supernatants of MSCs at a density of 1,000,000 cells per culture flask, cultured overnight in DMEM deprived of fetal calf serum and supplemented with 0.5% bovine serum albumin. After centrifugation at 2000 g for 20 min to remove debris, the cell-free supernatants were centrifuged at 100,000 g for 1 h at 4 °C, washed in serum-free medium containing DMEM 25 mM and subjected to a second ultracentrifugation under the same conditions. The MSC-MVs were stored at −80 °C. The protein content of MSC-MVs was quantified by Bradford assay. FACS analyses on isolated MVs were done as described [12]. Cytofluorimetric analyses showed the presence of several molecules such as CD44, CD29, and CD105 but not CD34 or CD45. Also, MSC-MVs were observed directly under a transmission electron microscope (JEM-1011; JEOL Ltd., Tokyo, Japan), and the photos were taken at a magnification of 10,000.

### MSCs hypoxia culture

The MSCs at a density of 1,000,000 cells per culture flask were treated in hypoxic conditions as previously described [11]. Briefly, MSCs and siHGF-MSCs were cultured for 3 days until confluent. Fresh complete medium was added before hypoxia induction. The MSCs were placed in a hypoxic incubator (BioSpherix, Ltd., Parish, NY, USA) for 24 h in an atmosphere of N_2_ (94.5%), O_2_ (0.5%) and CO_2_ (5%). After 24 h of hypoxic culture, supernatants were collected and centrifuged to remove debris, and stored at −80 °C.

### Co-culture protocol

First-passage endothelial cells were obtained from ScienCell Research Laboratories (Carlsbad, CA, USA). The endothelial cells were routinely characterized by the supplier. The cells were added into the upper chambers of 0.4 μm cell-culture inserts at a density of 50,000 cells per insert well and cultured in endothelial growth media (EGM)-2 (ScienCell Research Laboratories). The culture media was changed every 3 days, and the cells were used at passages 3–7 for all experiments. The endothelial cells were cultured at a density of 50,000 cells per well in six-well plates. After the endothelial cell reached confluence, the medium was changed with fresh culture medium or supernatants from MSC hypoxia culture (MSC-CM), MSC-MVs, or siHGF MSC-MVs.

### Endothelial cell permeability examination

Endothelial cells were seeded at 50,000 cells per insert well (0.4 μm pore size polyester membrane from Corning, Inc., Corning, NY, USA) and cultured for 1 to 3 days to allow the growth of a confluent monolayer. After the different groups received different treatments, the endothelial cell monolayers were treated with 100 ng/ml LPS for 6 h before testing for permeability. Paracellular and transcellular permeability were tested as described previously [9].

### Western blot analysis

After treatment, total protein was extracted from endothelial cells using RIPA lysis buffer supplemented with 1 mmol/L phenylmethanesulfonyl fluoride (PMSF, Beyotime Institute of Biotechnology, Shanghai, China), separated by 6% or 12% SDS-PAGE and transferred onto polyvinylidene fluoride membranes (Nanjing Lufei Biotechnology Co., Nanjing, China). Membranes were then blocked in phosphate-buffered saline-Tween (PBS-T) containing 5% milk for 2 h at room temperature and incubated at 4 °C overnight with primary antibodies against VE-cadherin (Cell Signaling, Danvers, MA, USA) or Occludin (Abcam, Cambridge, MA, USA). On the following day, the membranes were washed in PBS-T and incubated in peroxidase-conjugated secondary antibody (HuaAn Biotechnology, Hangzhou, China) for 1 h at room temperature. Immunoreactive bands were obtained using a chemiluminescence imaging system (ChemiQ 4800 mini, Ouxiang, Shanghai, China) with horseradish peroxidase.

### Immunofluorescence

A total of 5 × 10^4^ endothelial cells were seeded on 12-well culture plate and cultured for 1–3 d to allow for the growth of a confluent monolayer. After treatments, endothelial cells monolayers were treated with 100 ng/ml LPS before immunofluorescence. Endothelial cell were washed in cold PBS and fixed in 4% paraformaldehyde. Cells were incubated with 1% BSA in PBS for 30 min to block non-specific binding and then incubated with VE-cadherin antibody (Cell Signaling) at 4 °C overnight followed by incubation with FITC-conjugated goat anti-mouse IgG (Jackson Laboratory, Bar Harbor, MN, USA). Lastly, DAPI (4,6-diamidino-2-phenylindole) was used to stain nuclei. Single plain images of endothelial cells were obtained by microscope (Olympus, Tokyo, Japan) equipped with fluorescent illumination.

### Apoptosis and viability assays

Endothelial cells were cultured in 24-well plates until they reached 80% confluence.

Endothelial cell apoptosis was analysed using terminal deoxynucleotidyl transferase dUTP nick end labeling (TUNEL) assay following the manufacturer’s instructions. Briefly, cells were harvested and fixed in 4% paraformaldehyde, and cells were stained with a TUNEL Apoptosis Assay Kit (Nanjing Lufei Biotechnology Co., Nanjing, China). The images were observed with an Olympus microscope (Olympus). Cells were quantified by a pathologist based on five randomly selected high power fields (×400). The apoptosis index of cells, which was defined as the number of apoptotic cells divided by the total number of cells, was used to assess the severity of cells apoptosis. We adopted 3-(4,5-dimethylthiazol-2-yl)-2,5-diphenyltetrazolium (MTT) assay to detect endothelial cell viability. After the cells were treated, 60 μl MTT (Sigma-Aldrich, St., Louis, MO, USA) (5 mg/ml) was added to each well and incubated at 37 °C for 4 h. Two hundred microliters of dimethyl sulfoxide (DMSO; Sigma-Aldrich) was then added to the wells and incubated for 15 min. Finally, endothelial cell viability was analysed by measuring the absorbance of the sample at 570 nm and 630 nm.

### ELISA

After treatment, the supernatants were collected and centrifuged to remove debris. Interleukin (IL)-6 and IL-10 production was determined via an enzyme-linked immunosorbent assay (ELISA) using commercially available ELISA kits (ExCell Biology, Inc., Shanghai, China). ELISA was performed following the manufacturer’s instructions. All samples were measured in duplicates.

### Statistical analyses

Statistical analyses were performed using the SPSS 16.0 software package (SPSS Inc., Chicago, IL, USA). The results were presented as the mean ± standard deviation (SD). For comparisons of groups, one-way analysis of variance was used, followed by Tukey’s multiple comparison tests. *P* values less than 0.05 were considered statistically significant.

## Results

### Multilineage differentiation identification of MSC

The multipotent potential for differentiation along adipogenic, osteogenic, and chondrogenic lineages was determined by staining with oil red-O, alizarian red, or toluidine blue. The morphology of MSCs at the third passage (Fig. 1a, ×100) and multilineage differentiation capacities of MSCs, including adipogenic differentiation stained with oil red-O (Fig. 1b, ×200), osteogenic differentiation stained with alizarin red (Fig. 1c, ×200), and chondrogenic differentiation stained with toluidine blue (Fig. 1d, ×200), were observed with a microscope. Thus their identity as MSCs was verified.

### Identification of MSC-MVs by flow cytometry and electron microscope

**Fig. 2:**
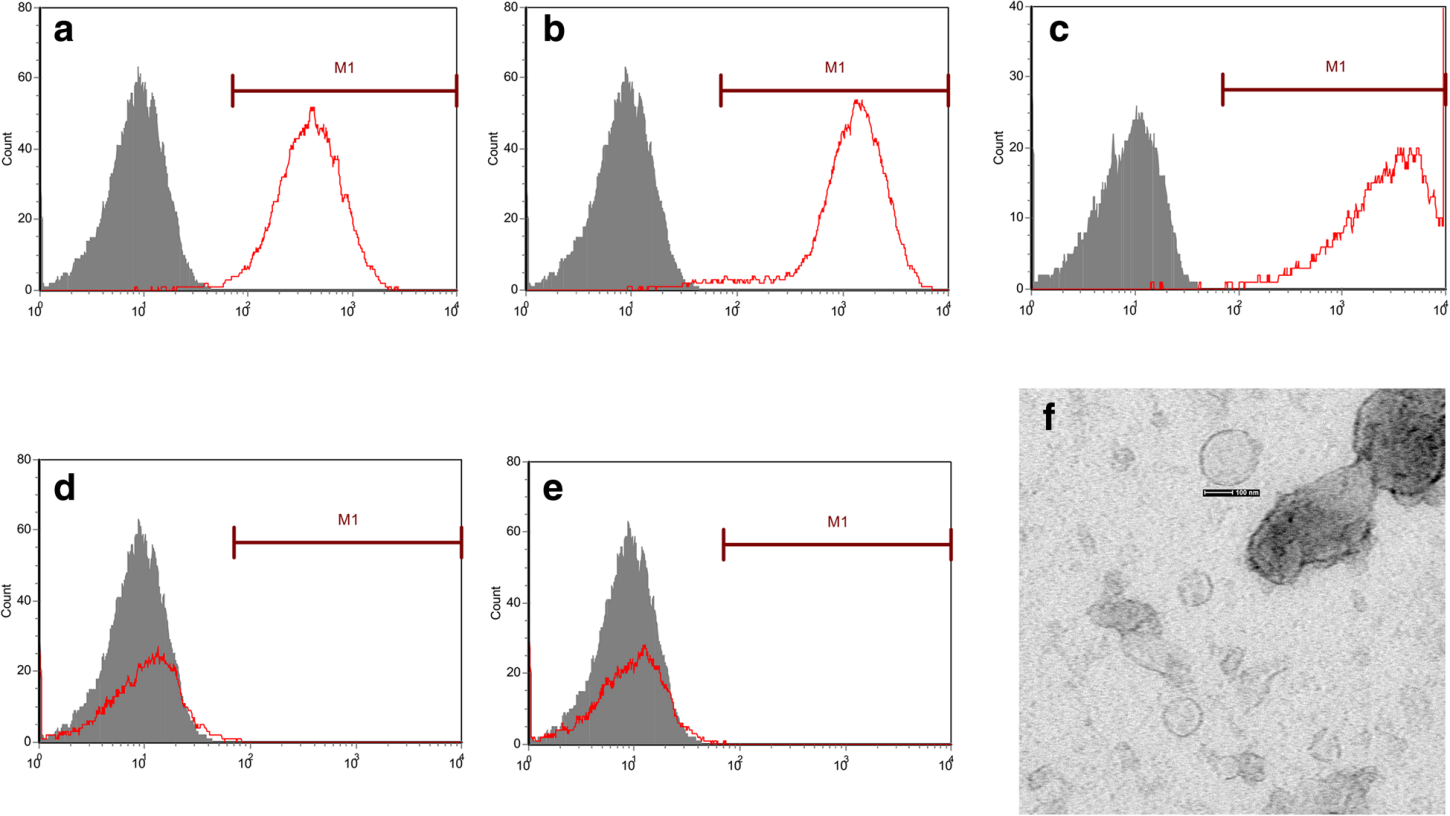
Identification of MSC-MVs by flow cytometry and electron microscopy. Cell surface markers of MSC-MVs, including CD34, CD44, CD105, CD29, and CD45, were analysed by flow cytometry. *Red lines* represent the isotype controls. Cytofluorimetric analyses showed the presence of several molecules such as CD44, CD29, CD105, but not CD34, CD45 (**a, b, c,** and **d**). Transmission and scanning electron microscopy performed on purified MSC-MVs indicated their spheroid morphology and confirmed their size. These conform to MSC-MVs characteristics (**f**)

MSC-MVs were identified by detecting surface phenotypes. Cell surface markers of MSC-MVs, including CD34, CD44, CD105, CD29, and CD45 were analysed by flow cytometry. Red lines represent isotype controls (Fig. 2). The company guaranteed that there were no ethical issues in using the MSC-MVs for experiments. Cytofluorimetric analyses showed the presence of several molecules such as CD44, CD29, CD105 but not CD34, CD45. Also, MSC-MVs were observed directly under a transmission electron microscope, and the photos were taken at a magnification of 10,000 (Fig. 2f). Transmission and scanning electron microscopy performed on purified MSC-MVs indicated their spheroid morphology and confirmed their size. These conform to MSC-MVs characteristics.

### The efficiency of HGF gene knockdown

**Fig. 3:**
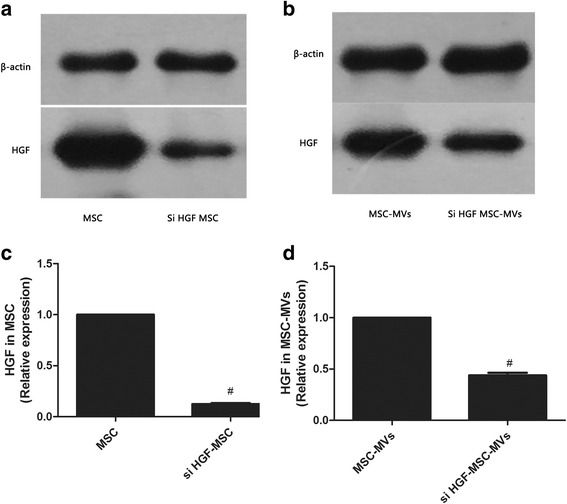
Measurements of HGF expression in MSC and MSC-MVs after HGF gene knockdown. The results showed that HGF expression in MSCs was decreased in the siHGF-MSC group compared to the MSC group (**a** and **c**, *p* < 0.05). Similarly, HGF protein expression in the MSC-MVs was decreased in the siHGF-MSC-MVs group compared to the MSC-MVs group (**b** and **d**, *p* < 0.05). (n = 3, *p < 0.05 vs. MSC or MSC-MVs group). *HGF* hepatocyte growth factor, *LPS* lipopolysaccharide, *MSC-MVs* mesenchymal stem cells microvesicles, *MSCs*, mesenchymal stem cells

In order to test the efficiency of HGF gene knockdown, HGF protein levels were detected by Western blotting. The results from Western blotting analysis showed that HGF expression in MSCs was decreased in the siHGF-MSC group compared to the MSC group (Fig. 3a and c, *p* < 0.05). Similarly, HGF protein expression in the MSC-MVs was decreased in the siHGF-MSC-MVs group compared to the MSC-MVs group (Fig. 3b and d, *p* < 0.05). The results showed we obtained a high efficiency of HGF gene knockdown.

### MSC-MVs reduced endothelial cell permeability via HGF

**Fig. 4:**
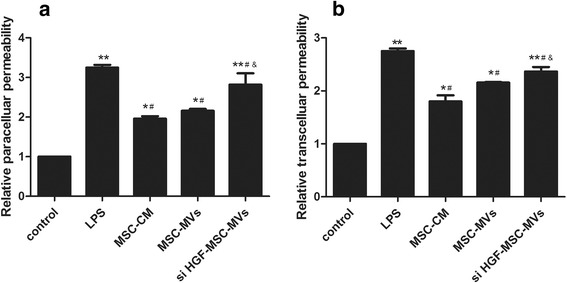
MSC-MVs reduced endothelial cell permeability via HGF. The results showed that treatment with MSC-MVs reduced hypoxia-induced endothelial paracellular and transcelluar permeability. However, the effect of treatment with MSC-MVs was significantly reduced after HGF gene knockdown. **a** The effect of MSC, MSC-MVs, and siHGF-MSC-MVs on relative endothelial paracellular permeability. **b** The effect of MSC, MSC-MVs, and siHGF-MSC-MVs on relative transcellular permeability. (n = 3, *p < 0.05, **p < 0.01 vs. control group; ^#^ *p* < 0.05 vs. LPS group; &*p* < 0.05 vs. MSC group). *HGF* hepatocyte growth factor, *LPS* lipopolysaccharide, *MSC-CM* mesenchymal stem cells conditioned medium, *MSC-MVs* mesenchymal stem cells microvesicles, *MSCs*, mesenchymal stem cells

There are two pathways regulating permeability across the endothelial barrier, paracellular and transcellular. It was necessary to reduce transcelluar permeability as well as paracelluar permeability to maintain endothelial barrier function in ALI [13]. Our previous research confirmed MSCs reduced LPS-induced endothelial paracellular and transcelluar permeability [14]. So we investigated the effect of MSC-MVs on endothelial paracelluar and transcelluar permeability. The results showed that endothelial paracelluar and transcelluar permeability were markedly increased after LPS stimulation. Treatment with MSC-MVs reduced LPS-induced endothelial paracellular and transcelluar permeability. However, the effect of treatment with MSC-MVs was significantly reduced after HGF gene knockdown (Fig. 4, *p* < 0.05). The results suggested that MSC-MVs lessen endothelial permeability by HGF.

### The effect of MSC-MVs on endothelial cell proliferation and apoptosis

**Fig. 5:**
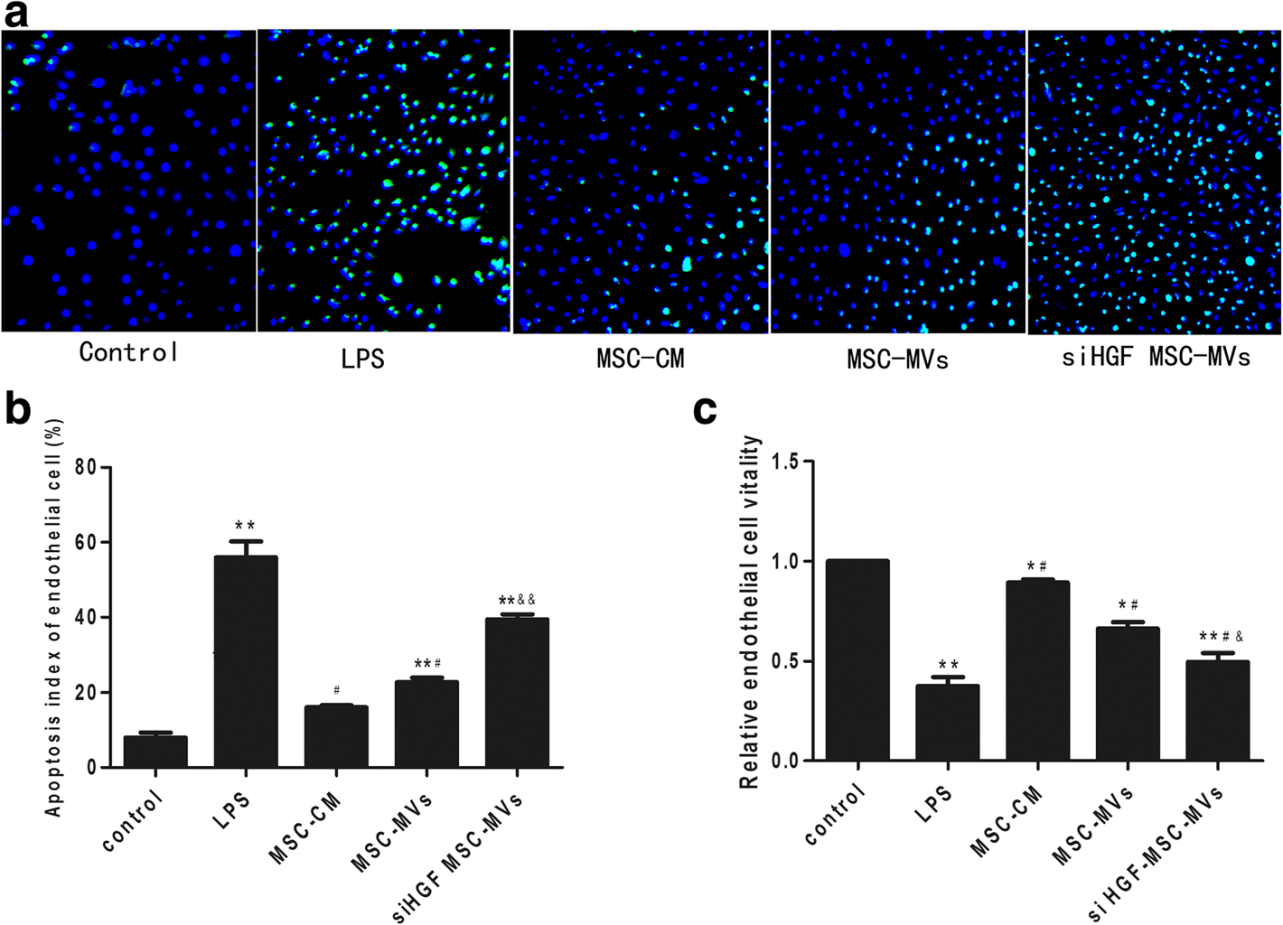
MSC-MVs decreased endothelial cell apoptosis and improved cell viability via HGF. Endothelial cell apoptosis was assessed using TUNEL assay kit. As **a** and **b** showed, the apoptosis index of endothelial cells were increased after LPS challenge, while treatment with MSC-CM or MSC-MVs treatment significantly reduced the apoptosis index of endothelial cells. Once again, the effect of MSC-MVs was significantly restrained after HGF gene knockdown. MTT assay confirmed that treatment with MSC-MVs restored cell viability to a greater extent than LPS stimulation alone. However, the effect of MSC-MVs was significantly inhibited after HGF gene knockdown (**c**). (n = 3, *p < 0.05, **p < 0.01 vs. control group; ^#^ *p* < 0.05 vs. LPS group; &*p* < 0.05 &&*p* < 0.01 vs. MSC-MVs group). *HGF* hepatocyte growth factor, *LPS* lipopolysaccharide, *MSC-CM* mesenchymal stem cells conditioned medium, *MSC-MVs* mesenchymal stem cells microvesicles, *MSCs*, mesenchymal stem cells

The survival of endothelial cell was evaluated using cell apoptosis and cell viability assays. As Fig. 5a and b showed, the apoptosis index of endothelial cells were increased after LPS challenge, while treatment with MSC-CM or MSC-MVs treatment significantly reduced the apoptosis index of endothelial cells. Once again, the effect of MSC-MVs was significantly restrained after HGF gene knockdown. MTT assay confirmed that treatment with MSC-MVs restored cell viability to a greater extent than LPS stimulation alone. However, the effect of MSC-MVs was significantly inhibited after HGF gene knockdown (Fig. 5c). Therefore MSC-MVs decrease endothelial apoptosis and induce proliferation in endothelial cells partly by HGF.

### MSC-MVs upregulated endothelial VE-cadherin and occludin protein expression via HGF

**Fig. 6:**
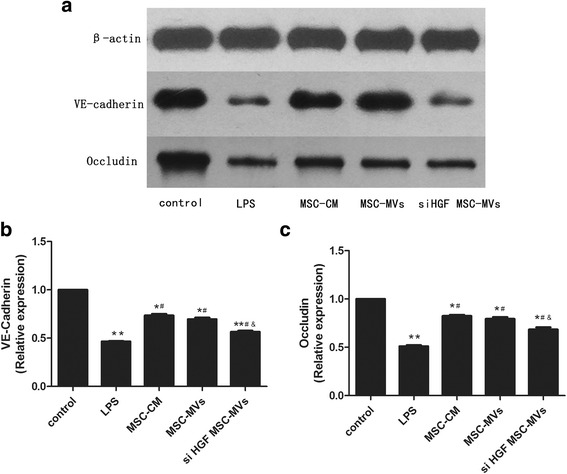
MSC-MVs upregulate endothelial VE-cadherin and occludin protein expression via HGF. **a, b** Image and quantitative results of VE-cadherin protein expression in the endothelial cells. **a, c** Image and quantitative results of occludin protein expression in the endothelial cells. The results showed that LPS stimulation of endothelial cell reduced the expression of VE-cadherin and occludin protein and that these effects were inhibited by MSCs and MSC-MVs. However, the effect of MSC-MVs was significantly restrained after HGF gene knockdown. (n = 3, *p < 0.05, **p < 0.01 vs. control group; ^#^ *p* < 0.05 vs. LPS group; &*p* < 0.05 vs. MSC-MVs group). *HGF* hepatocyte growth factor, *LPS* lipopolysaccharide, *MSC-CM* mesenchymal stem cells conditioned medium, *MSC-MVs* mesenchymal stem cells microvesicles, *MSCs*, mesenchymal stem cells

**Fig. 7:**
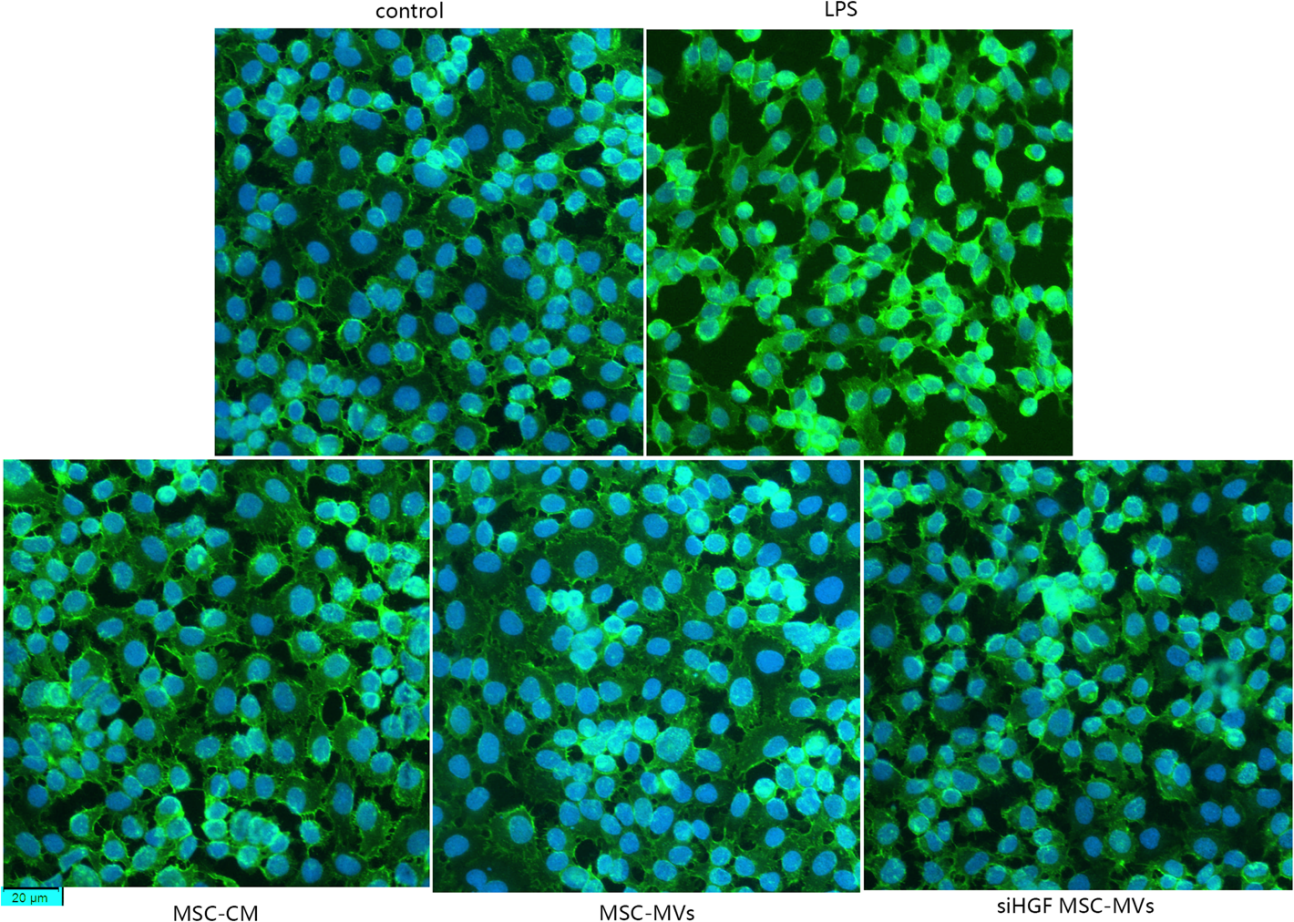
MSC-MVs restored remodelling of endothelial VE-cadherin by HGF. We further investigated the expression of VE-cadherin protein by immunofluorescence. The results showed that LPS induced dispersion of VE-cadherin. After MSC-MVs and endothelial cells co-culture, junctional localisation of VE-cadherin was partially restored. However, HGF gene knockdown in MSC-MVs caused VE-cadherin to be disrupted again

To illustrate the effect of HGF in MSC-MVs on endothelial permeability-associated protein, we examined endothelial VE-cadherin and occludin protein expression under the co-culture conditions. The results showed that LPS stimulation of endothelial cell reduced the expression of VE-cadherin and occludin protein and that these effects were inhibited by MSCs or MSC-MVs. However, the effect of MSC-MVs was significantly restrained after HGF gene knockdown (Fig. 6). We further investigated intercellular AJs protein by immunofluorescence. The results showed that LPS induced dispersion VE-cadherin. After MSC-MVs and endothelial cells co-culture, junctional localisation of VE-cadherin was partially restored. However, HGF gene knockdown in MSC-MVs caused VE-cadherin to be disrupted again (Fig. 7). The above results suggest that MSC-MVs restored the integrity of endothelial cell monolayers partly by HGF.

### The effect of MSC-MVs on IL-6 and IL-10 in the conditioned media of endothelial cells

Our previous research showed that HGF-expressing characteristic of MSC played an important part in regulatory inflammatory response [5]. Also research showed that MSC-derived MVs can inhibit in vitro a pro-inflammatory response to an islet antigenic stimulus in type 1 diabetes [15].

**Fig. 8:**
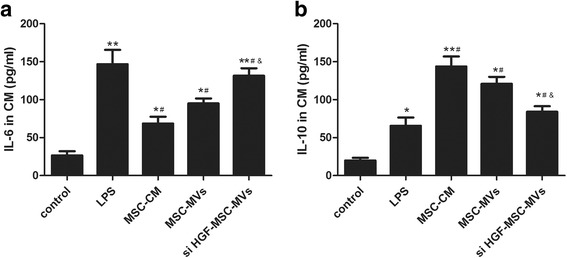
The effect of MSC-MVs on IL-6 and IL-10 in the conditioned media of endothelial cell. The results showed IL-6 level in the conditioned media of endothelial cells was markedly increased after LPS stimulation. After treatment with MSCs and MSC-MVs, IL-6 level in the conditioned media of the endothelial cells was significantly reduced, however, the effect of MSC-MVs was restrained after HGF gene knockdown (**a**). In contrast, treatment with MSC-MVs reduced the LPS-induced increase in IL-10 production in the conditioned media of endothelial cells. Similarly, the effect of MSC-MVs was restrained after HGF gene knockdown (**b**). (n = 3, *p < 0.05, **p < 0.01 vs. control group; ^#^ *p* < 0.05 vs. LPS group; &*p* < 0.05 vs. MSC-MVs group). *HGF* hepatocyte growth factor, *IL* interleukin, *LPS* lipopolysaccharide, *MSC-CM* mesenchymal stem cells conditioned medium, *MSC-MVs* mesenchymal stem cells microvesicles, *MSCs*, mesenchymal stem cells

Hence we also investigated the effect MSC-MVs on IL-6 and IL-10 production in the conditioned media of endothelial cells. The results showed IL-6 and IL-10 levels in the conditioned media of endothelial cells were markedly increased after LPS stimulation. After treatment with MSC conditioned medium (MSC-CM) or MSC-MVs, IL-6 level in the conditioned media of the endothelial cells was significantly reduced, however, the effect of MSC-MVs was restrained after HGF gene knockdown (Fig. 8a). In contrast, treatment with MSC-CM or MSC-MVs reduced the LPS-induced increase in IL-10 production in the conditioned media of endothelial cells. Similarly, the effect of MSC-MVs was restrained after HGF gene knockdown (Fig. 8b). Thus MSC-MVs regulate anti-inflammatory and inflammatory balance partly by HGF.

## Discussion

ALI is characterized by increased lung permeability, pulmonary oedema and diffuse inflammation and results in alveolar-capillary membrane disruption [16]. MSC-MVs have been shown to restore endothelial function [17]. However, the detailed mechanism by which MSC-MVs improve endothelial permeability remains unclear [18]. In the present study, we found that treatment with MSC-MVs significantly decreased LPS-induced endothelial permeability. This function of MSC-MVs is partially by HGF. By increasing the expression of the endothelial intercellular junction proteins VE-cadherin and occludin, HGF decreased endothelial apoptosis, induced endothelial cell proliferation and reduced IL-6 production and increased IL-10 production in the conditioned media of endothelial cells.

Pulmonary microvascular endothelial injury results in barrier dysfunction, which contributes to pulmonary oedema in ALI [19]. It is believed that two pathways regulate permeability across the endothelial barrier—the paracellular pathway and the transcellular pathway [20, 21]. The paracellular pathway mainly transports small molecules, while the transcellular pathway involves a vesicle-mediated transport of macromolecules across the endothelial barrier in a caveolae-dependent manner [22]. In our study, we used a hypoxia-induced endothelial cell permeability injury model. The results showed that the paracellular and transcellular permeabilities significantly increased at 24 h following hypoxia stimulation. Therefore, transcellular and paracellular permeability must be reduced to maintain endothelial barrier function in ALI.

Recent studies have indicated that the administration of MSCs improved endothelial permeability of ALI [23]. Interestingly, MSCs have been found to release microvesicles (MSC-MVs), which can be involved in cell-cell communication and the transfer of microRNA, protein or other signalling molecules [24, 25]. Zhu et al. [8] have reported that MSC-MVs had the same therapeutic effect as the MSCs themselves in *E. coli* endotoxin-induced ALI in mice through the transfer of KGF microRNA, which decreased endothelial permeability. In our study, we found that the treatment with MSC-MVs reduced LPS-induced paracellular and transcellular permeabilities. Moreover, the therapeutic effect of MSC-MVs was similar to the MSCs themselves.

HGF secreted by MSCs is a key factor associated with endothelial permeability [9, 13]. Studies have shown that HGF mRNA present in MVs derived from stem cells was delivered into cells and translated into the HGF protein as a mechanism of HGF’s induction of cell differentiation and growth [11]. Thus, we assume that HGF derived from MSC-MVs might have key roles in the regulation of endothelial permeability by MSC-MVs. In this study, we found that MSC-MVs decreased endothelial paracellular and transcellular permeability, however, the effect was significantly restrained after HGF gene knockdown. These results indicated that HGF derived from MSC-MVs has a key role in the regulation of endothelial permeability by MSC-MVs.

HGF appears in the lung circulation under pathological conditions, such as ALI, and exhibits continuous barrier protective effects on human pulmonary endothelial cells [26, 27]. However, its detailed mechanism is unclear. Some studies have suggested that HGF-induced endothelial permeability effects on endothelial cells have been associated with the increased expression of the adherens junction protein VE-cadherin and the tight junction protein occludin [28]. Endothelial permeability is mainly determined by adherens and tight junctions [29]. Our results showed that LPS stimulation of endothelial cells reduced the expression of VE-cadherin and occludin proteins and it also induced endothelial apoptosis, and restrain endothelial cell proliferation. Moreover, these effects were inhibited by MSCs and MSC-MVs. However, the effect of MSC-MVs was significantly inhibited after HGF gene knockdown. Thus, HGF derived from MSC-MVs regulated endothelial permeability by remodelling of the endothelial junction.

One limitation of our study should be noted. Our study suggested that HGF derived from MSC-MVs has a key role in the regulation endothelial permeability by MSC-MVs. However, the study was done in vitro. Our future study will employ further research in vivo to verify the role of HGF in the stabilization of endothelial barrier function by MSC-MVs.

## Conclusions

In summary, we demonstrated that treatment with MSC-MVs reduced LPS-induced paracellular and transcellular permeabilities. The therapeutic effect of MSC-MVs was similar to the MSC-CM themselves. Moreover, the effect of MSC-MVs was significantly restrained after HGF gene knockdown, which showed that HGF derived from MSC-MVs has a key role in the regulation of endothelial permeability. Additionally, possible mechanisms of HGF-regulated endothelial permeability involve remodelling of the endothelial junction, reduction of endothelial apoptosis, and induction of endothelial cell proliferation.

## Abbreviations

AJ: Adherens junction
ALI: Acute lung injury
ELISA: Enzyme-linked immunosorbent assay
HGF: Hepatocyte growth factor
IL: Interleukin
KGF: Keratinocyte growth factor
LPS: Lipopolysaccharide
MSC-CM: Mesenchymal stem cells conditioned medium
MSC-MVs: Mesenchymal stem cells microvesicles
MSCs: Mesenchymal stem cells
MVs: microvesicles
PMVECs: Pulmonary microvascular endothelial cells
TUNEL: Terminal deoxynucleotidyl transferase dUTP nick end labeling
